# G-Protein coupled receptors: answers from simulations

**DOI:** 10.3762/bjoc.13.106

**Published:** 2017-06-02

**Authors:** Timothy Clark

**Affiliations:** 1 Computer-Chemie-Centrum, Department of Chemistry and Pharmacy, Friedrich-Alexander-University Erlangen-Nuernberg, Naegelsbachstr. 25, 91052 Erlangen, Germany

**Keywords:** computer-aided drug design, GPCR, metadynamicxs, molecular dynamics

## Abstract

Molecular-dynamics (MD) simulations are playing an increasingly important role in research into the modes of action of G-protein coupled receptors (GPCRs). In this field, MD simulations are unusually important as, because of the difficult experimental situation, they often offer the only opportunity to determine structural and mechanistic features in atomistic detail. Modern combinations of soft- and hardware have made MD simulations a powerful tool in GPCR research. This is important because GPCRs are targeted by approximately half of the drugs on the market, so that computer-aided drug design plays a major role in GPCR research.

## Introduction

Evolution is a unique optimization mechanism. Firstly, it stops optimizing as soon as an acceptable solution is reached. There is no evolutionary pressure for elegance, simplicity or even effectiveness above the critical threshold. Secondly, because evolution always starts with what is already available, it reuses successful designs again and again in slightly modified forms. This is the case for the most common means of communicating across cell walls in eukaryotes, G-protein coupled receptors (GPCRs). GPCRs span the cell membrane and generally complex switching ligands from the extracellular medium in order to effect changes in the G-protein signaling system inside the cell. There are many variations on the scenario, some of which will be outlined below. Approximately 800 GPCRs are encoded in the human genome [1], earning them the label “*The Evolutionarily Triumphant G-Protein Coupled Receptor*” [2]. Their functions are myriad, from olfactory and visual receptors to pure signaling systems that govern cell function. Malfunction of GPCRs is prevalent in human diseases, so that approximately half (estimates vary between 30 and 60%) of marketed drugs target GPCRs. Furthermore, cancer cells can misuse existing GPCRs to ensure their own survival and prevalence [3]. It is therefore not surprising that GPCRs are the subject of a vast research effort that was recognized by the award of the 2012 Nobel Prize in Chemistry to Robert Lefkowitz [4] and Brian Kobilka [5].

**Figure 1:**
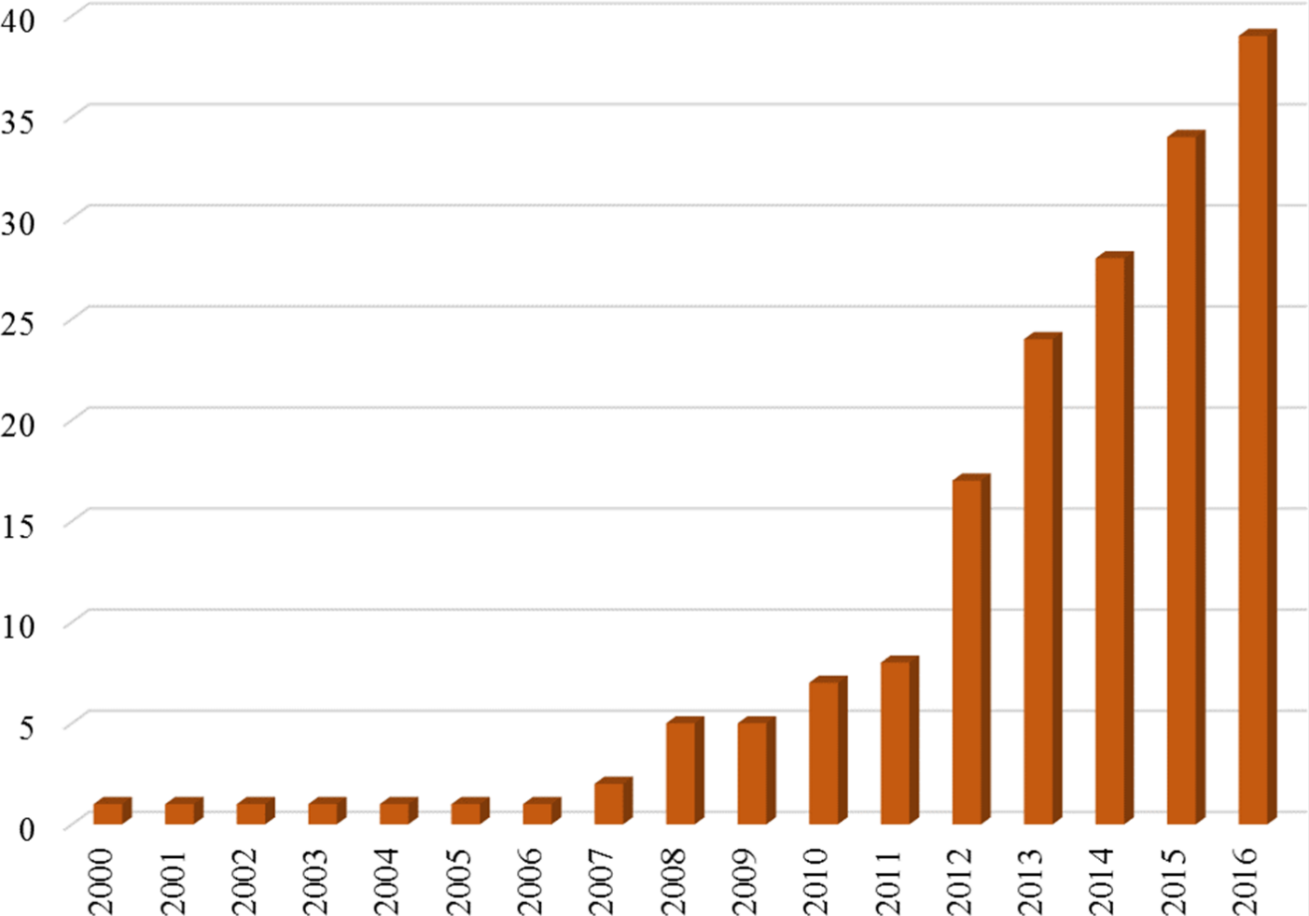
The cumulative number of different GPCRs for which X-ray structures were available in a given year. The data represent a total of 174 structures on 91 ligand–receptor complexes for 39 different receptors. The data are taken from http://gpcrdb.org/structure/statistics (2nd February 2017).

The experimental research to date on GPCRs represents a landmark in scientific achievement because of the complexity and experimental intractability of GPCRs themselves. Structural information that can be used as the basis for simulations is most important for the purposes of this review. Ultimately, structures at atomistic resolution are needed to decipher the intimate details of the modes of action of GPCRs. X-ray crystallographic studies on GPCRs are, however, fraught with difficulties [6]. The structure of rhodopsin, the first GPCR X-ray structure, was published in 2000 [7], was not followed by the second, the β2-adrenergic receptor, until 2007 [8]. Figure 1 shows the growth in the number of GPCR structures from 2000–2016. After a very slow start, structures for on average six new receptors per year have been becoming available in the last five years. Each of these structures is a significant experimental achievement, so that the low number of structures being published represents the output of a major worldwide research effort to obtain structures for receptors that unfortunately require considerable ingenuity (and luck) to obtain suitable crystals for X-ray crystallography [6].

Not only has the paucity of available structures hampered investigations, however, GPCRs can exist in active or inactive conformations and in binary complexes with ligands or intracellular binding partners (IBPs, G-proteins or β-arrestin) or in ternary complexes with a ligand and an IBP. Thus, many structures would be necessary in order to obtain a complete atomistic picture of the mode of action of the GPCR. A further problem is that we need structures that correspond to the receptors in their natural surroundings as they occur and function in nature. Proteins, especially membrane-bound ones, do not necessarily crystallize in their biologically active structures and the measures needed to obtain suitable GPCR crystals tend to increase the diversity between the natural environment and the crystal.

These measures are needed to overcome some inherent problems in crystallizing GPCRs. These problems may arise from flexible or non-polar regions of the GPCR, especially intracellular loop 3 (IL3), that do not form the rigid, specific interactions needed for crystallization. Such problems are sometimes overcome by truncating the flexible termini, complexing the GPCR with antibody fragments [9], or by replacing IL3 with a stable, polar fragment such as the T4 lysozyme [10–11] or other suitable protein fragments [12]. Other techniques used to obtain GPCR crystals include mutations to enhance the thermal stability [13], solubilization with custom detergents [14] or in conjunction with high-affinity ligands, which promote one stable conformation. These techniques are discussed in far more detail in reference [6].

One further problem in GPCR structure determination is to obtain crystals in which the GPCR is in the active conformation. The active conformation could be stabilized at low pH with detergents for opsin/rhodopsin [15–16] and the critical IL3 was resolved in both cases. However, the loop conformation was stabilized by intermolecular contacts in the crystals, as was later shown spectroscopically [17]. This conformation is only stable in ternary complexes with G-proteins in the natural systems but G-proteins are not stable enough for crystallization. The solution to this problem has been to use the variable domains of camelid antibodies, which are generally designated protein nanobodies, as a surrogate for the G-protein [18]. This technique will be discussed in the context of the simulations below. Note, however, that the opsin/rhodopsin structures [15–16] used so-called high-affinity peptides to mimic the G-protein. It is likely that these proteins behave more like the native G-protein than the protein nanobodies.

## Review

### General GPCR structure

**Figure 2:**
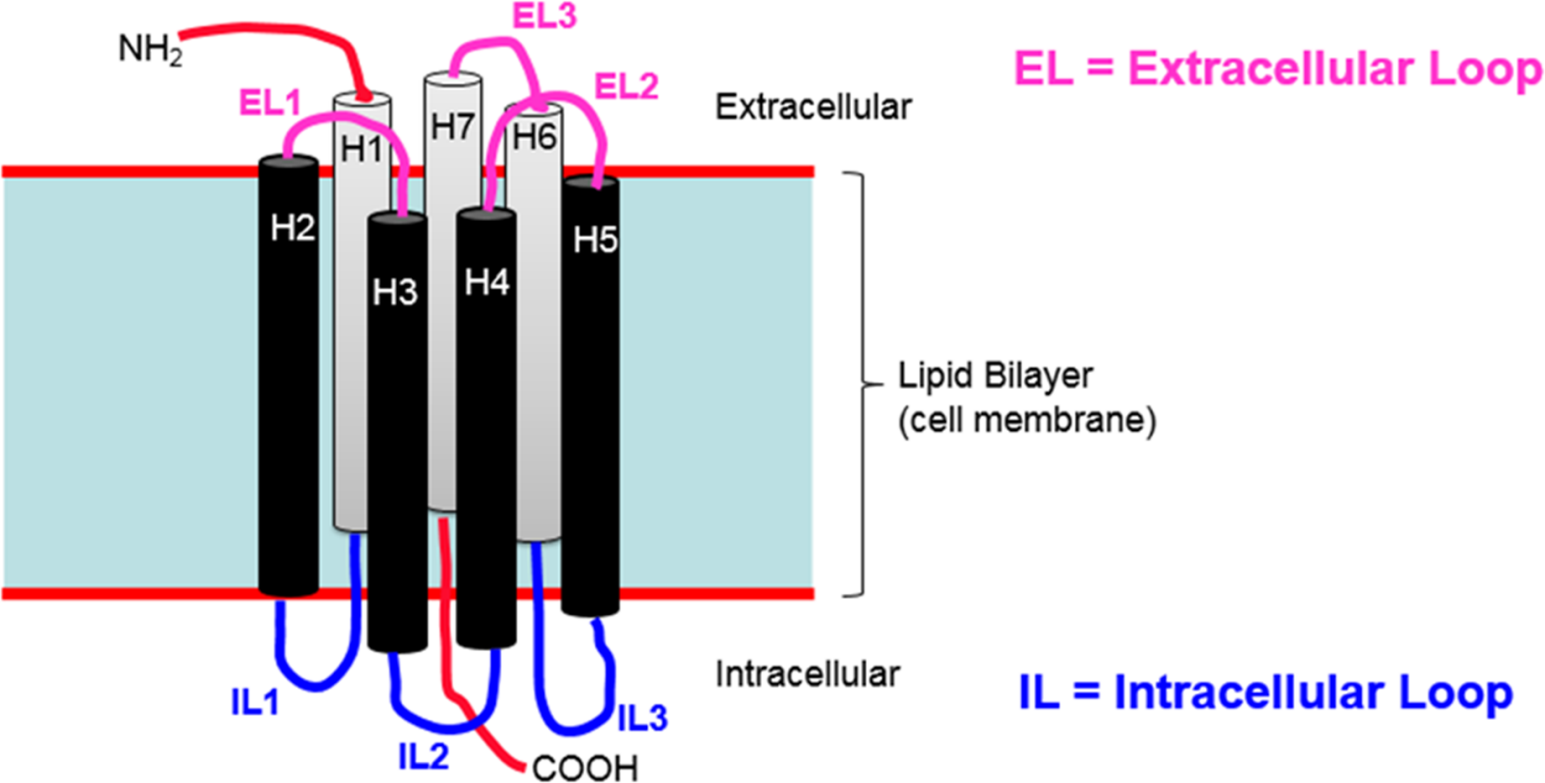
A schematic diagram of the general structure of GPCRs. Most GPCRs also contain an eighth helix, H8, at the C-terminus (not shown in the Figure).

Figure 2 shows a schematic diagram of the general structure of GPCRs.

GPCRs consist of seven α-helices that span the membrane between the extra- and intracellular sides. The N-terminus is extracellular and the C-terminus intracellular. The helices are connected by three intracellular loops (IL1, H1-H2; IL2, H3-H4 and IL3, H5-H6) and three extracellular ones (EL1, H2-H3; EL2, H4-H5 and EL3, H6-H7). The extracellular loops are often involved in ligand recognition and binding, whereas the intracellular ones interact with the IBPs, usually a G-protein. The activation process involves switching of the binding on the intracellular side of the receptor, as outlined below.

### Mechanism of G-protein signaling

**Figure 3:**
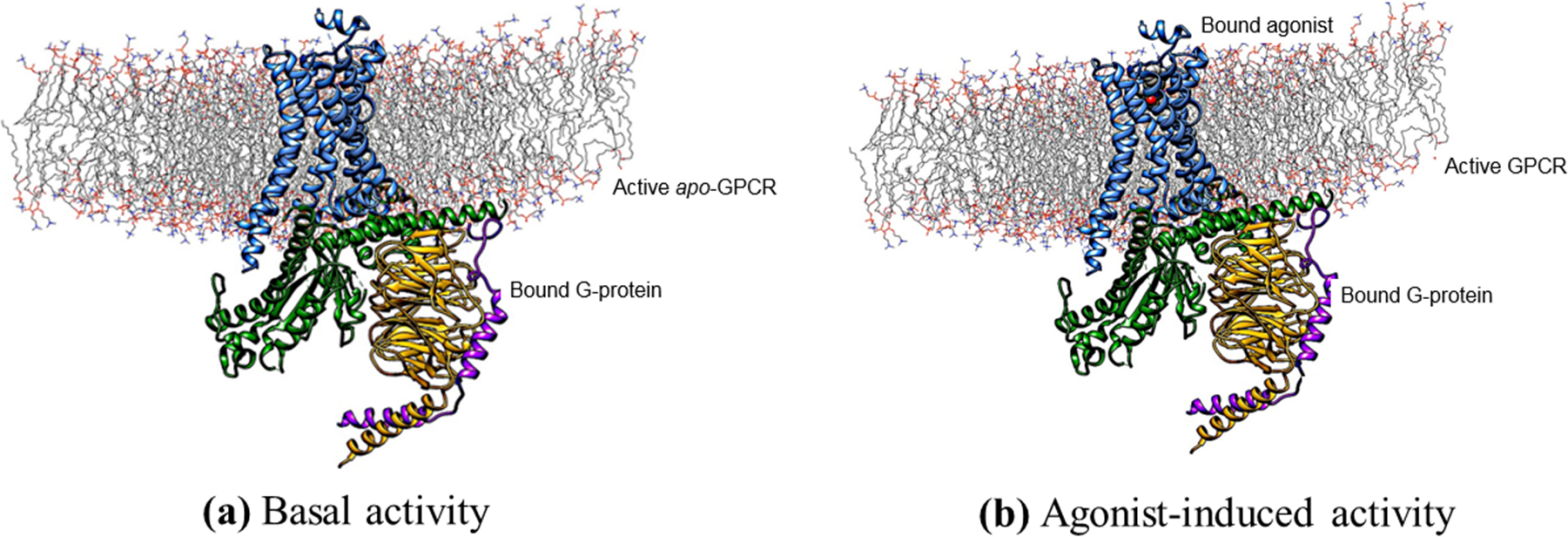
(a) The inactive state of a GPCR: No ligand is bound. The α-subunit of the G-protein is shown in green, β in yellow and γ in magenta. In this state, the GPCR exhibits basal activity. This figure assumes pre-association of the G-protein to the receptor. (b) Fully activated GPCR. Both an agonist ligand and the G-protein are required for full activation. The structures shown are based on homology models.

**Figure 4:**
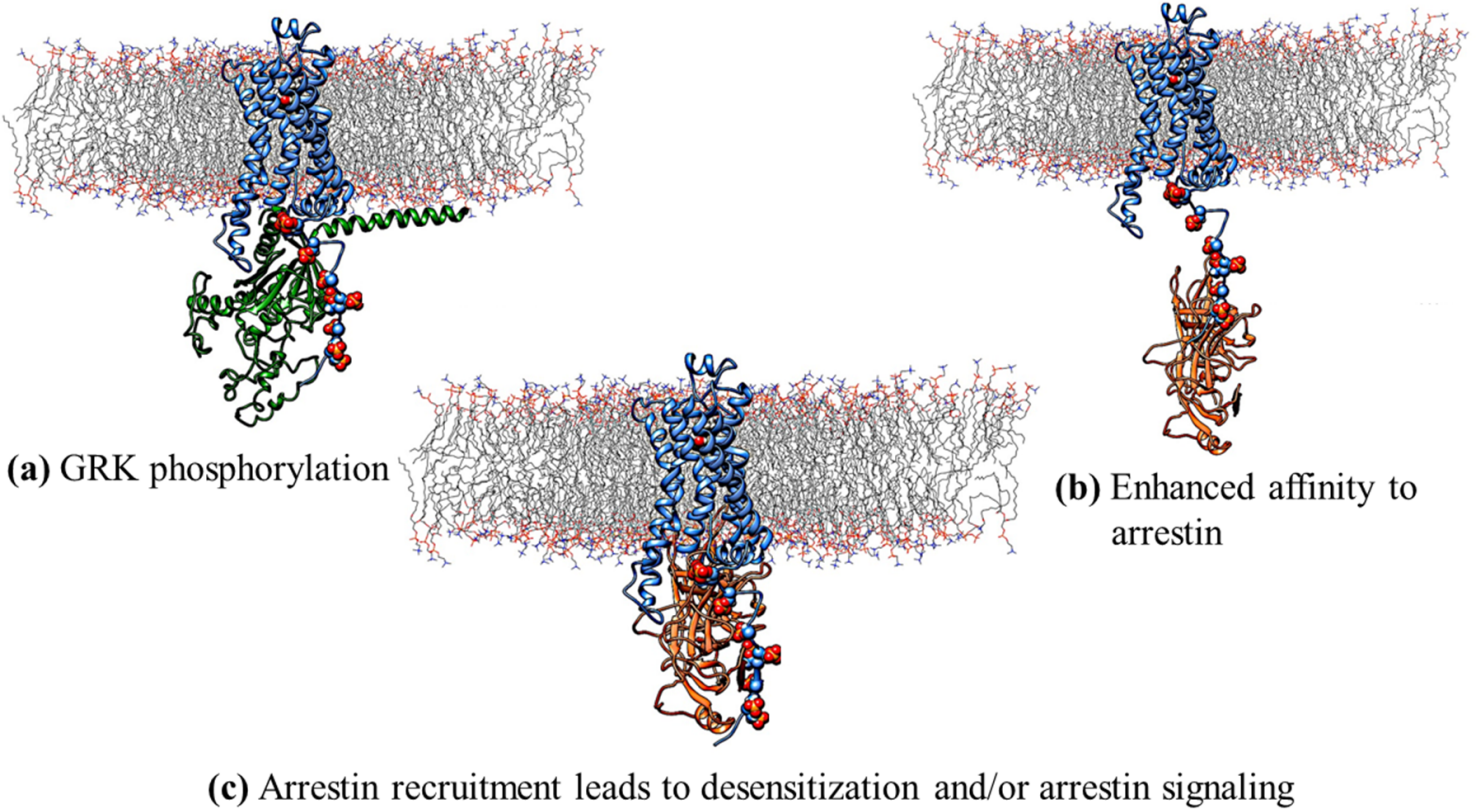
(a) After activation of the GPCR and dissociation of the β/γ subunits, IL3 and the C-terminus of the GPCR is phosphorylated by G-protein regulating kinases (GRKs). (b) Arrestin is recruited by the phosphorylated tail. (c) The fully bound arrestin can lead either to internalization of the receptor (inactivation) or to a separate arrestin signaling path. This Figure represents only one of many possibilities, which have been reviewed by Tobin [19].

Figure 3 and Figure 4 show modeled structures to illustrate the mechanisms of signaling in GPCRs. In the simulations discussed below, the G-protein is represented only by the α-subunit, which binds directly to the GPCR.

In the ligand-free state shown in Figure 3a, no agonist ligand is bound. The G-protein is bound to the intracellular side of the receptor. In this state, the GPCR exhibits its basal activity, which can range from completely inactive to significantly active. Figure 3b shows the fully activated complex, which requires both an agonist ligand and the G-protein. On activation, bound guanosine diphosphate (GDP) in the G-protein is replaced by the triphosphate (GTP) and the α-subunit separates from β/γ. The separated G-protein subunits migrate to effectors in the nearby membrane, where GTP is hydrolyzed to GDP and the signaling cascade initiated.

GPCRs are normally deactivated by β-arrestin, as shown in Figure 4. After activation and dissociation of the β/γ subunit, IL3 and the C-terminus of the GPCR are phosphorylated at serine and threonine residues (Figure 4a). This phosphorylation allows the recruitment of β-arrestin (Figure 4b), which can then form a strongly bound complex with the receptor (Figure 4c). This complex can lead to internalization of the receptor (its removal from the cell wall) or to an independent β-arrestin signaling pathway. This inactivation process is subject to very many variations, depending on the GPCR, and internalization may be reversible if the phosphorylated residues are hydrolyzed within the cell.

### GPCR modeling and simulation

GPCRs are such important pharmaceutical targets that homology models of various receptors were constructed [20] almost as soon as the rhodopsin structure [7] became available. Even though it has played a major role in the determination of the mechanisms of action of GPCRs [21–22] and was the only structure available, in general, rhodopsin was not considered the ideal template for GPCR drug targets. This was because of both its relatively low similarity to medicinal targets [23] and its distance from them in the G-protein phylogenetic tree [1]. Later, when the β2-adrenergic receptor structure was published [8] it was concluded that homology models would play an increasingly important role in computer-aided drug design (CADD) [24]. With hindsight, this conclusion was perhaps a little optimistic. However, the situation has changed considerably in the last five years. Not only are more (and more relevant) GPCR structures available, but modern protein force fields have attained a level of reliability that makes them truly predictive in most drug-design scenarios; it was found five years ago that “*the calculation error is comparable to the uncertainty in the experimental comparison*” [25]. The error in this case refers to the ability of the force field to reproduce peptide and protein conformations determined using calibrated Karplus equations in conjunction with NMR experiments. The more important development is, however, the power of modern hardware. For years, 10 ns simulations were state of the art. These simulations had little relevance for real biological systems, which are generally far slower. It became evident from studies on transcription factors that simulations often require a simulation induction period of several hundred nanoseconds to a microsecond before they undergo important conformational changes [26]. This is possibly because the starting structures are usually taken from X-ray crystal structures or homology model derived from them. As the force fields have been optimized to reproduce X-ray structures, they likely have a kinetic bias that hinders rearrangement from typical starting structures. Nonetheless, the simulations were able to predict whether the receptor was induced (i.e., allows transcription) or not [26], in contrast to X-ray structures in which the allosteric rearrangement was overlaid by crystal-packing effects.

Today’s combinations of hard- and software allow routine simulations of several μs, which means that homology models can be equilibrated long enough for them to adopt what is probably a biologically relevant structure. These simulations are, however, still not long enough to investigate processes such as the binding or unbinding of GPCR ligands and IBPs. In these cases, we must resort to enhanced-sampling techniques, the most effective of which in our hands has proven to be metadynamics [27]. As outlined below, modern variations of metadynamics allow very effective use of massively parallel supercomputers in order to investigate ligand binding and unbinding and transitions between active and inactive receptor conformations. Indeed, the power of modern simulations is such that we must revisit the relationship between simulations and experiment, especially for GPCRs.

### Experiment and simulations

The traditional, and very persistent, view of the relationship between experiments and simulations is that, if the latter cannot reproduce the former, the simulations are inaccurate. This may in many cases be true, although, subjectively at least, the accuracy of simulations is closely related to that of experiments in the same research area. That is not, however, the main point with GPCR-research. We are faced with an extremely difficult research area in which every result is valuable. Some experimental results are obtained under extremely difficult conditions and may not be reproducible. A major aspect of this discussion, however, has to do with the relationship between the biological system and the necessarily modified objects studied in experiments. GPCRs are flexible, sensitive proteins that, because of their biological function, react sensitively to perturbations. Given the reliability of protein force fields pointed out above, it should be clear that it is often possible to simulate systems that are closer to the biological situation than the crystals used to obtain X-ray structures. An early example of this is the fact that crystal-packing forces are large enough to change the induction state of the tetracycline repressor [26]. GPCR simulations often also show geometric rearrangements after several hundred nanoseconds, which suggests that the simulation is perhaps switching to a conformation closer to the biologically relevant one than the X-ray structure. Also, the simulations do not need the modifications outlined above for obtaining suitable crystals; they can be performed for the original biological system.

Thus, simulations can reasonably be expected in many cases to give a closer picture of the biological situation than some experiments. Another point is, however, important and does not result in competition between simulations and experiment; simulations can provide information that is so far not available from experiment. This is an important but still largely unrecognized aspect of GPCR research. Even the most skeptical about the accuracy and relevance of simulation can accept at least the role of simulations to point towards detailed mechanistic aspects that suggest specific experimental tests. Of course, the simulations must be validated as far as possible by comparison with experiment but without forgetting that the error limits for the experiments are often comparable to those of the simulations. For instance, free binding energies from simulations that agreed with experimental ones by, say, less than 0.5 kcal mol^–1^ would mean that not only the simulations but also the experiments are far more accurate than we expect.

In the following, GPCR simulations that provide atomistic details of GPCR activation mechanisms will be described. These are mostly from our own work but also include some landmark simulations from elsewhere.

### Binary and ternary complexes

The ternary complex model [28] and experimental findings [29] suggest that both an agonist ligand and a bound G-protein are necessary in order to activate GPCRs. It is therefore significant that the first molecular dynamics (MD) simulations of a ternary GPCR complex were reported only four years ago [30]. Such simulations are now commonplace and the comparison between binary ligand–receptor and ternary complexes has become a valuable tool in GPCR research.

### Activation mechanism

The first simulations to demonstrate a binding pathway for ligands approaching a GPCR from the extracellular medium were reported for the β1- and β2-adrenergic receptors in 2011 [31]. Notably, these simulations were performed on Anton, a specially constructed computer for MD simulations [32], and were unconstrained, so that they simulated the ligand-binding process without enhanced sampling on a time scale of several μs. Later simulations of the same type revealed a mechanism for allosteric modulation for the muscarinic M2 receptor [33]. Most importantly, though, long unconstrained simulations were able to demonstrate the deactivation of an active conformation of the β2-adrenergic receptor (taken from the X-ray structure) [18] in binary ligand–receptor complexes. These unconstrained simulations lay the foundations for more targeted ones that use enhanced-sampling techniques to determine, for instance, the activation mechanism of the muscarinic M2 receptor [34].

### Free energies of binding by metadynamics

Very long timescale MD simulations can be performed on specialized hardware such as Anton [25] but are less effective on more conventional massively parallel supercomputers because the simulations only scale up to a relatively limited number of CPUs or GPUs [35]. Luckily, of the many enhanced-sampling techniques [35], modern variations of metadynamics [27] can make very effective use of massively parallel hardware. Briefly, metadynamics enhances the sampling in MD simulations by adding small Gaussian destabilizing potentials at positions that the simulations has already visited enough. “Positions” need to be defined in terms of a small number of geometrical variables (the collective variables, CVs) that are relevant (e.g., as a reaction coordinate) for the process being studied. In this respect, the relatively fixed orientation of the GPCR in the membrane allows us to define a generally applicable CV perpendicular to the plane of the membrane [36]. This general CV describes the binding process of ligands approaching from the extracellular medium remarkably well. This in itself would not make the simulations effective on massively parallel supercomputers but the use of many replicas at the same time to enhance the sampling (multi-walker metadynamics) [37] further allows many simulations to be carried out in parallel, and thus makes excellent use of massively parallel hardware. The final enhancement to the simulations is to apply a so-called funnel constraint [38] that limits the sampling in the extracellular solution, where it is not necessary [36].

In our context, the most important advantage of metadynamics is that it gives a free-energy profile of the process being simulated [27]. This means that we can obtain complete free-energy profiles along the binding path for both ligands and IBPs [39]. This, in turn, allows us to validate the simulations by comparison with experimental free energies of binding obtained from measured binding constants. This comparison turned out to be an unqualified success; the simulated binding energies for 23 different binary and ternary complexes comprising five different receptors and 13 different ligands gave a root mean square deviation of 0.8 kcal mol^–1^ [36]. In contrast to other techniques used to predict binding energies, the simulations deliver an excellent agreement with the experiment (the correlation line has a slope of 0.99 and an intercept of zero, with R^2^ = 0.81), rather than simply correlating well. Remarkably, the ligands span a wide range of efficacies; in 10 cases, they act as agonists, in 11 as antagonist and twice as partial agonists. One key to this success is that the simulations were able to identify the most stable binding site of several alternatives in each case.

### Multiple binding sites

We have often observed that in quantitative structure–activity relationships (QSAR) for GPCRs, agonists give far better results than antagonists [40]. Metadynamics simulations on the vasopressin receptor [41] revealed the reason for this behavior. As also found previously in unbiased simulations of the β2-adrenergic receptor [31], ligands can occupy more than one binding site along the binding path. In the case of vasopressin, a cyclic peptide hormone, the simulations revealed three different sites, the conventional orthosteric one that activates the ligand, an “intermediate” and a “vestibule” site. Significantly, antagonists bind to one of the alternative sites more strongly than to the orthosteric one. Of pharmacological importance is the fact that antagonists bind to different sites in the two subtypes of the vasopressin receptor investigated, so that a general QSAR that encompasses agonists and antagonists for both receptors would need to consider all three sites [41].

Multiple binding sites along the binding path are common in GPCRs. The human chemokine receptor CXCR3, for instance, exhibits distinct alternative binding sites that can be occupied simultaneously by competing ligands, which explains contradictory experimental results obtained in competition experiments [42]. Multiple binding sites have also been found for the β2-adrenergic, muscarinic M2 and μ-opioid receptors [36,39].

### Functional bias

For those GPCRs that can activate both G-protein and β-arrestin pathways, some ligands may exhibit a functional bias and activate one or other of the two alternative paths. Metadynamics simulations have proven to be able to determine the bias, or lack of it, by considering the change in ligand-binding free energy between the binary ligand–receptor complex and the alternative ternary complexes with either the G-protein α-subunit or β-arrestin. We define two free-energy differences:


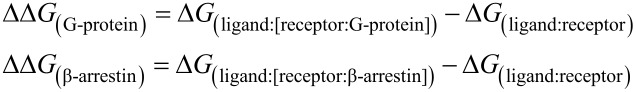


The ligand bias can be determined from these energies according to Table 1 [39].

**Table 1:** Scheme for determining the bias of GPCR ligands according to the calculated changes in ligand-binding free energies [39].

ΔΔ*G*_(G-protein)_	ΔΔ*G*_(β-arrestin)_	Ligand bias

negative	negative	unbiased agonist
negative	positive	G-protein biased agonist
positive	negative	arrestin biased agonist
positive	positive	unbiased reverse agonist
≈zero	≈zero	neutral antagonist

Thus, the simulations allow not only the calculation of the free energy of binding for unknown ligands but also the functional bias.

## Conclusion

The simulations described are extremely compute-intensive; they have been performed on SuperMUC [43] with grants totaling 85 million CPU hours and using thousands of cores per simulation. However, considering the progress being made constantly in computer soft- and hardware, such simulations will become routine within a decade or less. Two take-home messages are important.

Firstly, the simulations can provide information not available (yet) from experiments. This is because the experiments are very difficult, because they must be performed in many cases on modified receptors and because atomistic details are available from very few experimental sources. Thus, simulations should be accepted as valuable tools in GPCR research.

Secondly, even given their very high computational cost, simulations may even now be a viable alternative to experiment for determining binding constants (= free energies of binding) and ligand bias. The simulations are predictive and can therefore be used in prospective computer-aided drug design.
